# 717 Management of Gynecologic Involvement in Stevens-Johnson Syndrome (SJS)/ Toxic Epidermal Necrolysis Syndrome (TENS)

**DOI:** 10.1093/jbcr/irad045.192

**Published:** 2023-05-15

**Authors:** Rachel Knapp, Marcia Ciccone, Haig Yenikomshian, Justin Gillenwater

**Affiliations:** Keck School of Medicine of USC, Los Angeles, California; University of Southern California, Los Angeles, California; University of Southern California, Los Angeles, California; Keck Medicine of USC, Los Angeles, California; Keck School of Medicine of USC, Los Angeles, California; University of Southern California, Los Angeles, California; University of Southern California, Los Angeles, California; Keck Medicine of USC, Los Angeles, California; Keck School of Medicine of USC, Los Angeles, California; University of Southern California, Los Angeles, California; University of Southern California, Los Angeles, California; Keck Medicine of USC, Los Angeles, California

## Abstract

**Introduction:**

Stevens-Johnson Syndrome (SJS)/ Toxic Epidermal Necrolysis Syndrome (TENS) are rare and potentially life-threatening dermatologic conditions that require interdisciplinary management (Shanbhag et al., 2020). One of the most painful and traumatic is vaginal mucosal involvement. Gynecologic complications have been historically undermanaged, and lack of acute intervention can lead to dyspareunia, adhesion formation, and increased risk for vaginal cancers (Kaser et al., 2011). Unfortunately, treatment is variable but typically involves use of vaginal dilators, which can be painful, and often traumatic for both patient and provider. The purpose of this study was to analyze the utilization of these treatments and to observe any sequelae from gynecological involvement.

**Methods:**

Retrospective chart review was completed for all female patients with a diagnosis of SJS/TENS who were admitted to a single center burn unit from 2015-2022. Trends in age, gynecologic involvement, wound care strategies, and outcomes were assessed.

**Results:**

Twenty-nine patients met inclusion criteria. These patients ranged from 9-82 years of age with a mean of 43 (STD 21). Ten percent died in the hospital, 14% were transferred to another hospital, and the remaining patients were discharged to home or a rehabilitation facility. Of these 29 SJS/TENS patients, 59% had gynecologic involvement of which 52% received gynecologic interventions. Gynecologic interventions included use of vaginal dilators (67%), topical steroids (60%), antifungal ointment (33%), and menstrual suppression (13%). For pediatric populations, patient discomfort and family concerns limited pelvic exam and vaginal interventions. Accommodations for pediatric patients included completion of examination and treatment under anesthesia and education of parents about the treatment process. While 2 patients were confirmed to have no vaginal adhesions in follow up appointments, documentation of future vaginal complaints was not available for most patients.

**Conclusions:**

Gynecologic management is an important part of SJS/TENS treatment with wide variability in practice patterns not only nationally but at a single setting institution. Outcomes from treatment versus non treatment with steroids +/- dilation is equivocal. Providers must be better at screening for symptoms in outpatient follow up. When forming new algorithms, special consideration among pediatric populations and invasive management needs to be considered.

**Applicability of Research to Practice:**

There is wide practice variability on the gynecological management of SJS/TENS patients with variable outcomes. Longitudinal studies on outpatients would better help create algorithms for patient management.

